# Exploring Urinary Tract Injuries in Gynecological Surgery: Current Insights and Future Directions

**DOI:** 10.3390/healthcare13151780

**Published:** 2025-07-23

**Authors:** Martina Arcieri, Margherita Cuman, Stefano Restaino, Veronica Tius, Stefano Cianci, Carlo Ronsini, Canio Martinelli, Filippo Bordin, Sara Pregnolato, Violante Di Donato, Alessandro Crestani, Alessandro Morlacco, Fabrizio Dal Moro, Lorenza Driul, Giuseppe Cucinella, Vito Chiantera, Alfredo Ercoli, Giovanni Scambia, Giuseppe Vizzielli

**Affiliations:** 1 Department of Obstetrics and Gynaecology, Azienda Sanitaria Universitaria Friuli Centrale, Ospedale Santa Maria della Misericordia, 33100 Udine, Italy; martina.arcieri@asufc.sanita.fvg.it (M.A.); lorenza.driul@uniud.it (L.D.); giuseppe.vizzielli@uniud.it (G.V.); 2 Obstetrics and Gynaecology, Department of Medical Area (DAME), University of Udine, 33100 Udine, Italy; cuman.margherita@spes.uniud.it (M.C.); tius.veronica@spes.uniud.it (V.T.); bordin.filippo@spes.uniud.it (F.B.); pregnolato.sara@spes.uniud.it (S.P.); 3 PhD School in Biomedical Sciences, Gender Medicine, Child and Women Health, University of Sassari, 07100 Sassari, Italy; 4 Unit of Gynecology and Obstetrics, Department of Human Pathology of Adult and Childhood “G. Barresi”, University of Messina, 98122 Messina, Italy; stefano.cianci@unime.it (S.C.); canio.martinelli@unime.it (C.M.); alfredo.ercoli@unime.it (A.E.); 5 Department of Woman, Child and General and Specialized Surgery, University of Campania “Luigi Vanvitelli”, 81100 Naples, Italy; carlo.ronsini@unicampania.it; 6 Sbarro Institute for Cancer Research and Molecular Medicine and Center of Biotechnology, College of Science and Technology, Temple University, 1900 N 12 St, Philadelphia, PA 19122, USA; 7 Department of Gynecological, Obstetrical and Urological Sciences, “Sapienza” University of Rome, 00184 Rome, Italy; violante.didonato@uniroma1.it; 8 Department of Urology, Santa Maria della Misericordia Hospital, 33100 Udine, Italy; alessandro.crestani@asufc.sanita.fvg.it; 9 Department of Surgery, Oncology and Gastroenterology (DISCOG), University of Padova, 35122 Padova, Italy; alessandro.morlacco@unipd.it (A.M.); fabrizio.dalmoro@unipd.it (F.D.M.); 10 Department of Gynecologic Oncology, Istituto Nazionale Tumori—Fondazione “G. Pascale” IRCCS, 80131 Naples, Italyvito.chiantera@istitutotumori.na.it (V.C.); 11 Department of Women, Children and Public Health Sciences, Fondazione Policlinico Universitario Agostino Gemelli IRCCS, 00168 Rome, Italy; giovanni.scambia@policlinicogemelli.it; 12 Facoltà di Medicina e Chirurgia, Università Cattolica del Sacro Cuore, 20123 Rome, Italy

**Keywords:** urinary injury, hysterectomy, management, genitourinary fistula

## Abstract

Iatrogenic urinary tract injury is a known complication of pelvic surgery, most commonly occurring during gynecological procedures. The bladder and ureters are particularly vulnerable due to their close anatomical proximity to the uterus. Urinary tract damage can result from various mechanisms, including laceration, ligation, and thermal injury. Incidence rates vary according to the affected organ and surgical type; bladder injuries occur in 0.24% of benign and 0.4–3.7% of oncologic surgeries, whereas ureteral injuries are reported in 0.08% of benign and 0.39–1.1% of oncologic procedures. Timely diagnosis is essential for effective management. When detected intraoperatively, the injury can often be repaired immediately. Surgical treatment options vary depending on the specific nature and location of the bladder or ureteral damage. Delayed diagnosis can significantly impact the patient’s quality of life, increasing the risk of severe complications such as genitourinary fistulas. This narrative review aims to summarize current evidence on the diagnosis, prevention, and treatment of urinary tract injuries occurring during gynecological surgery. It evaluates risk factors, incidence, management, complications, and prevention strategies for iatrogenic bladder and ureteral injuries. Additionally, it highlights the innovative role of artificial intelligence in preventing urologic damage during gynecological procedures. The relevant literature was identified through a structured search of the PubMed database using predefined keywords related to gynecological surgery and urinary tract injury.

## 1. Introduction

Iatrogenic injuries of the urinary tract mainly occur during pelvic surgeries, including urologic, colorectal, and gynecological procedures [1]. Gynecological surgeries account for more than 50% of all urological injuries, while urological procedures contribute approximately 30% and general surgical procedures account for 5% to 15% of cases [2,3,4,5] [Table 1].

During gynecological surgeries, the ureters and bladder are particularly vulnerable due to their close anatomical relationship with the uterus. Ureteral injuries may result from laceration, ligation, devascularization, or electrosurgical trauma. In contrast, bladder injuries are most frequently caused by sharp dissection or inadvertent incision [1].

Hysterectomy is the most widely performed gynecological procedure and carries the highest risk of urological complications. It is performed for both benign and malignant indications. Factors that alter normal pelvic anatomy, such as deep infiltrating endometriosis, uterine enlargement, and pelvic adhesions, significantly increase the risk of urinary tract injury [6]. The incidence of urinary tract injury during gynecologic laparoscopy for benign indications is approximately 0.33%, with variations depending on whether the bladder or ureter is involved [6]. Among benign indications, surgery for endometriosis demonstrates the highest incidence of ureteral injury, estimated at 0.4% [6]. Endometriosis is a chronic inflammatory condition that promotes adhesion formation, complicating the identification of the bladder and ureters. In advanced disease, endometrial implants may infiltrate these structures, further elevating the risk of urinary tract injury during surgical excision [7].

Conversely, a global systematic review analyzing 1.74 million women found that, during hysterectomy for malignancy, the bladder injury rate rose as high as 997 per 100,000 procedures (0.997%), and ureteric injury climbed up to 814 per 100,000 (0.814%) [4]. The risk and incidence of urologic complications are substantially higher in radical hysterectomy compared to procedures for benign conditions, primarily due to the complex surgical maneuvers required [8]. These include accessing the ureteral tunnel, extensive dissection of periureteral tissues, and bladder mobilization.

In gynecological malignancies, tumor infiltration disrupts normal tissue planes, significantly increasing the complexity of surgical dissection [9]. Additionally, preoperative radiotherapy can further alter pelvic anatomy by inducing fibrosis and tissue fragility, thus making surgical dissection more challenging [10]. In advanced cases, extensive surgical intervention is often required due to the involvement of multiple pelvic organs, necessitating a multidisciplinary approach and consequently elevating the risk of urinary tract injury. The incidence of urological complications is particularly high in patients with cervical cancer and in those with a history of prior urologic surgery [11]. As highlighted in the literature, these complex procedures should be performed by gynecologic surgeons specializing in oncologic pathology and with advanced training to minimize the risk of urological complications [12].

**Figure 1:**
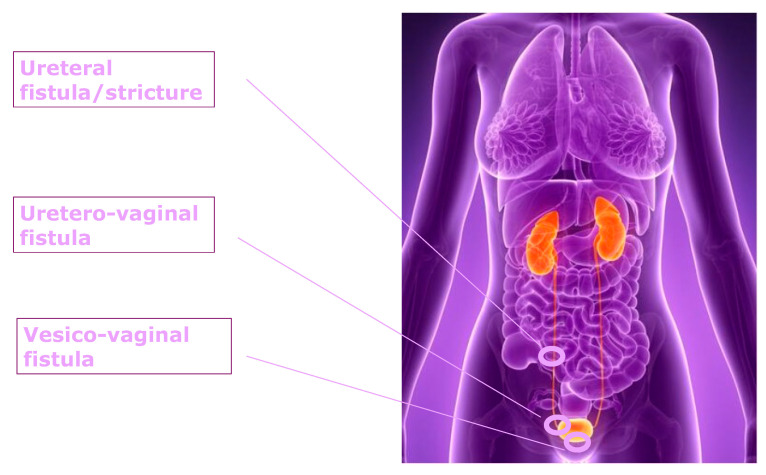
Urinary tract fistulas. This schematic illustration shows the most common types of urinary fistulas involving the ureter and bladder in the female pelvis. The fistulas are named in the order in which they appear in the figure; from top to bottom: ureteral fistula, which is an abnormal connection between the ureter and an adjacent organ such as the uterus or bowel; uretero-vaginal fistula, a pathological tract between the ureter and the vagina; and vesicovaginal fistula, a direct communication between the bladder and vagina.

Genitourinary fistulas are uncommon but serious complications that most frequently arise following hysterectomy. Among these, vesicovaginal fistulas are the most prevalent, with an estimated incidence of 0.8%, followed by ureterovaginal fistulas, which occur in approximately 0.16% of cases [13,14]. Although rare, these complications have a profound impact on patients’ quality of life, affecting not only physical health but also psychological and social well-being [15] (Figure 1).

Urinary tract injuries may be detected either intraoperatively or postoperatively, with significant implications for patient morbidity [16]. Early intraoperative recognition is essential for optimal management and can substantially reduce the risk of adverse outcomes. Various strategies have been developed to minimize urological injury and facilitate timely detection [17].

This narrative review aims to guide the diagnosis and management of urinary tract injuries in gynecological surgery by summarizing the main risk factors and incidence rates of iatrogenic bladder and ureteral injuries, critically appraising current clinical management approaches and their associated complications, evaluating preventive strategies designed to minimize urinary tract injury, and exploring the emerging role of innovative technologies, including artificial intelligence, in enhancing surgical safety and patient outcomes. By integrating recent high-impact literature and global epidemiological data, this review offers a comprehensive and critical overview intended to guide clinicians and researchers in improving patient care within this challenging surgical field.

## 2. Methods

This narrative review was conducted following the Scale for the Assessment of Narrative Review Articles (SANRA) guidelines to ensure methodological transparency and reliability.

A thorough literature search was performed in PubMed up to March 2025 to identify relevant studies on iatrogenic urinary tract injuries associated with gynecological surgery. The search strategy involved combinations of keywords, including “iatrogenic urinary tract injury” AND “gynecological surgery” AND (“bladder injury” OR “ureteral injury” OR “diagnosis” OR “prevention” OR “treatment” OR “artificial intelligence”). The search was limited to articles published in English, with no restrictions on publication date.

Inclusion criteria encompassed original research articles, systematic reviews, meta-analyses, and case reports addressing the diagnosis, prevention, or treatment of urinary tract injuries in gynecological surgery. Publications exploring the role of innovative technologies—particularly artificial intelligence—in surgical planning or intraoperative guidance were also included. Exclusion criteria were non-English publications, editorials, commentaries, letters to the editor, abstracts without full text, and studies not directly related to urinary tract injury in gynecologic surgical contexts.

Two authors independently screened titles and abstracts, followed by full-text assessment to determine eligibility. Any discrepancies were resolved through discussion.

Selected studies were reviewed in full, and key data regarding study type, clinical focus, and technological applications were extracted. The findings were narratively synthesized, structured around recurring themes and innovations, to provide an integrated overview of the current state of evidence. Although no formal quality assessment tool was applied, particular attention was paid to the methodological clarity and clinical relevance of the included studies.

## 3. Bladder Injury

Bladder injury may occur at multiple stages of gynecological surgery, with critical steps including bladder dissection during hysterectomy, adhesiolysis, and trocar insertion in the suprapubic region [18].

During benign gynecological procedures, the incidence of bladder injury is approximately three times higher (0.24%) than that of ureteral damage [6]. This increased risk is primarily due to the necessary dissection of the vesicouterine peritoneum to perform colpotomy, whereas the ureteral roll maneuver is generally not performed in hysterectomies for benign conditions. Two major risk factors for bladder injury during hysterectomy for benign indications have been identified: previous cesarean delivery [19] and the presence of deep infiltrating endometriosis [20]. A history of cesarean section often leads to the formation of fibrotic adhesions between the bladder and the lower uterine segment. These adhesions hinder the identification of the bladder–uterine septum, complicating the downward bladder dissection required for colpotomy.

Additionally, the influence of the surgical approach for hysterectomy in benign conditions on the incidence of bladder injury has been investigated, with varying results reported across studies. Sandberg’s meta-analysis, which compared only laparoscopic and vaginal hysterectomy, found no significant difference in bladder injury rates between these two approaches [21]. Conversely, a German study comparing minimally invasive techniques with laparotomy reported higher bladder injury rates in total vaginal hysterectomy and laparoscopically assisted hysterectomy (0.235% and 0.20%, respectively) compared to abdominal and total laparoscopic hysterectomy (0.16% and 0.12%, respectively) [22]. In vaginal hysterectomy, the bladder base is exposed by applying traction to the uterus via the vaginal route, a critical step during which iatrogenic bladder injury may occur. Notably, over the past decade, a decline in the use of the vaginal approach has been observed [23]. This trend may contribute to higher rates of bladder injury associated with vaginal hysterectomy, possibly due to reduced familiarity and confidence with the procedure on the part of the surgeon.

In contrast, hysterectomy performed for malignant pathology is associated with higher rates of bladder injury, ranging from 0.4% to 3.7% [24]. Incidence rates vary according to cancer type, with bladder injury reported in approximately 1.1% of endometrial cancer cases [25] and 2.2% of cervical cancer cases [26]. The increased frequency of bladder injury in radical hysterectomy (RH), especially for cervical malignancies, is largely attributed to anatomical distortion caused by tumor infiltration. Radical hysterectomy is a complex procedure often required in gynecologic oncology. Bladder injury during RH may also result from inadvertent damage to the hypogastric nerve, which plays a critical role in detrusor muscle function. Therefore, nerve-sparing techniques, such as isolating the hypogastric nerve via development of the Okabayashi space, are essential to preserve bladder function [27]. Furthermore, the surgical approach influences bladder injury rates in malignant cases, with minimally invasive radical hysterectomy demonstrating higher incidence of bladder damage compared to the abdominal approach [28].

### 3.1. Diagnosis, Management, and Treatment

Approximately 85% of bladder injuries are identified intraoperatively [16]. Typical intraoperative signs include visible defects in the bladder wall, visualization of the Foley catheter through the injury site, presence of gas bubbles in the urine bag during laparoscopy, macroscopic hematuria, and urine leakage [29]. When direct visualization of the injury is not possible, but suspicion remains high, the bladder condition can be assessed by retrograde filling with saline mixed with blue dye. The appearance of colored fluid within the pelvic cavity confirms the presence of a bladder wall disruption. Cystoscopy is a valuable tool for intraoperative detection, enabling immediate visualization and, if needed, prompt repair of bladder injuries. Bladder injuries may involve various anatomical regions, with the bladder dome being most commonly affected [15]. Injuries involving the bladder trigone are considered more severe due to their proximity to the ureteral orifices, making assessment of the lesion’s distance from these orifices critical. Cystoscopy facilitates evaluation of both the size and precise location of the injury in relation to the trigone and ureteral orifices [30].

When not detected intraoperatively, bladder injuries are typically diagnosed in the postoperative setting, based on clinical signs such as peritonitis, sepsis, abdominal pain, hematuria, decreased urinary output, or elevated serum creatinine levels [29]. The recommended imaging methods for confirming bladder injury in the postoperative setting include computed tomography (CT) and cystography [29,31].

The treatment of bladder injury primarily involves surgical repair followed by catheter drainage [32]. However, there is no clear consensus in the literature regarding the optimal suture type and size for bladder wall repair. Various suture materials have been employed, with polyglactin and poliglecaprone absorbable sutures being the most commonly used due to their minimal tissue reactivity and absorption within approximately 21 days [33,34]. Although less commonly used, olain catgut sutures have been shown to reduce bladder tissue reaction and have longer tensile strength duration compared to polyglactin, despite the bladder’s rapid healing capacity [34]. In recent years, barbed sutures have emerged as a feasible and safe alternative for bladder repair. Their key advantage lies in their knotless design, utilizing barbs to achieve self-anchorage to the tissue, thereby reducing operative time compared to conventional sutures [35,36]. According to recent reviews, the most frequently used suture sizes are 2–0 and 3–0 [34]. Bladder wall closure can be performed in one or two layers, using either interrupted or continuous suturing techniques. The choice of technique typically depends on the surgeon’s preference, as no definitive evidence favors one method over another [18,34,37].

There is also no consensus regarding the optimal duration of postoperative catheterization. Traditionally, catheterization ranges from 5 to 14 days; however, a recent systematic review reported durations varying widely from 1 to 42 days without significant differences in complication rates [33]. In cases where the bladder injury involves the trigone, management may necessitate ureteral stenting of one or both ureters or surgical reimplantation [1,34].

### 3.2. Vesicovaginal Fistula

A vesicovaginal fistula (VVF) is an abnormal communication between the bladder and vagina, resulting in involuntary and continuous urinary leakage through the vaginal canal. The diagnosis of VVF is primarily clinical, based on the presence of continuous urinary leakage through the vagina [38]. When clinical suspicion remains high despite inconclusive initial examination, a dye test can be performed [39]. This involves instilling a saline solution mixed with blue dye into the bladder via a Foley catheter, followed by the patient performing a cough or a Valsalva maneuver. The presence of blue dye on a vaginal tampon confirms the diagnosis of VVF. For more precise evaluation of the fistula, cystoscopy, and CT are utilized [40]. Hysterectomy, performed for either benign or malignant indications, represents the most common surgical cause of VVF [38,41]. The risk of genitourinary fistula formation increases notably following radical hysterectomy due to tissue hypovascularization, which predisposes the area to fistula development [42].

VVFs are traditionally classified by size (small < 0.5 cm, medium 0.6–2.4 cm, and large > 2.5 cm) and type (i.e., a single, non-irradiated, small tissue fistula or complex, i.e., a medium or large, irradiated, multiple, or recurrent fistula) [42]. According to Goh’s classification [Table 2], additional important parameters include the distance from the external urethral meatus to the distal edge of the fistula and the fistula’s diameter, enhancing the clinical utility of the classification system [43].

VVF can be treated via vaginal, abdominal, or laparoscopic approaches, with or without robotic assistance. Optimal treatment requires careful consideration of the fistula’s characteristics. The vaginal approach includes techniques such as the Latzko procedure [44] and layered closure [42], both associated with low morbidity rates and a reported success rate of 93.82%, according to a recent systematic review [13]. The Latzko technique [42] involves exposing the vaginal wall about 1 cm around the fistula without removing the fistulous tract at the bladder level. A multilayered, overlapping closure is then performed separately on the bladder, vesicovaginal fascia, and vagina wall [44]. In contrast, with the layered closure technique, the fistula is removed by separating the bladder from the vaginal mucosa and underlying fascia for about 1.5 cm [45]. The bladder is then closed in two layers using 3–0 or 4–0 absorbable sutures—the first layer positioned outside the mucosa and the second overlapping it [45]. The abdominal approach may be performed employing either a transvesical (O’Conor Technique) or an extravesical one (Bivalve technique), both involving bladder incision but differing in their anatomical access [40].

The transversal approach utilizes an abdominal incision at the bladder dome, allowing visualization and excision of the fistula, followed by bladder closure perpendicular to the vaginal repair [40]. The extravesical technique involves a vertical incision from the bladder dome to the fistula tract, with separate two-layer closures of the vaginal wall and bladder [42]. Laparoscopic and robot-assisted laparoscopic repairs are increasingly recognized as safe and effective, with comparable success rates and reduced morbidity compared to open abdominal surgery. Complex fistulas may benefit from combined surgical approaches—for instance, a combination of vaginal and abdominal techniques [46] or vaginal and vesicoscopic methods [47]. Despite the variety of described surgical routes, no universal consensus exists on the optimal treatment strategy. This is largely due to the absence of standardized fistula classification systems, heterogeneous reporting of success rates, and surgeon preference playing a significant role in approach selection. A meta-analysis focusing on VVF repair after benign gynecological surgery noted that most cases are managed transvaginally, despite there being insufficient evidence to support superiority of this approach [13]. A more recent systematic review confirmed that the choice of surgical route remains at the surgeon’s discretion, with no significant difference in outcomes among techniques [48]. Moreover, the impact of surgical experience on repair success has not been adequately studied, highlighting a need for further research [48]. Tissue interposition between the vagina and bladder is often employed to enhance vascularization and lymphatic drainage, thereby promoting healing and reducing infection and recurrence risks [40]. In abdominal repairs, omental flaps, epiploic appendages, or peritoneal flaps have been utilized for vaginal repairs, the Martius flap—a labial flap containing bulbocavernosus muscle and fatty tissue—being the most commonly employed [49]. However, a recent randomized controlled trial reported that the use of interpositional flaps does not significantly impact fistula repair success rates, challenging their historical role as a standard adjunct [50].

### 3.3. Prevention

The most straightforward method for preventing bladder damage is to ensure bladder emptying via catheterization during at-risk procedures [34]. For example, preoperative bladder emptying in hysterectomy reduces iatrogenic urinary tract injury risk [34]. However, controlled bladder filling can sometimes aid surgical dissection by improving identification of cleavage planes [34]. Additional strategies to minimize the risk of bladder injury include the careful use of electrosurgery near the bladder to avoid thermal damage, performing sharp dissection over blunt techniques to preserve anatomical structures, and applying cephalad traction using a uterine manipulator [34]. During laparoscopic hysterectomy, in particular, the combination of cephalad traction on the uterus via the manipulator and counter-traction on the vesicouterine peritoneum facilitates safe bladder mobilization [18].

While cystoscopy has a well-established role in diagnostic urology, its utility as an intraoperative screening tool following hysterectomy remains controversial [51]. According to Chi et al., routine cystoscopy after hysterectomy reduces the likelihood of delayed recognition of bladder injuries [52]. However, a meta-analysis has shown that universal cystoscopy does not significantly reduce the incidence of postoperative bladder injury in hysterectomies performed for benign indications [53]. This finding is supported by a recent U.S.-based study, which reported no difference in the rate of postoperative bladder injuries between patients who underwent cystoscopy and those who did not—regardless of whether the hysterectomy was performed for benign or malignant conditions. Notably, even after intraoperative cystoscopy, there remains a 0.27% incidence of delayed urinary tract injury [17]. Moreover, cystoscopy may fail to detect certain injuries, such as small bladder perforations and thermal damage—mechanisms that are particularly relevant in minimally invasive procedures. Although cystoscopy is generally considered a low-risk procedure, it has been associated with an increased rate of urinary tract infections when performed during hysterectomy [53]. Additionally, its use prolongs operative time by approximately 9 min, thereby increasing surgical costs [51,54].

In final analysis, the routine use of cystoscopy after hysterectomy remains controversial. While it may facilitate early detection of injuries, it does not significantly reduce postoperative complications and is associated with increased infection risk, added operative time, and higher costs. Selective use in high-risk patients may represent a cost-effective compromise.

## 4. Ureteral Injury

Among gynecological surgeries, hysterectomy is associated with the highest risk of ureteral injury due to the ureter’s anatomical course and the nature of the surgical maneuvers involved. Most iatrogenic injuries occur in the distal ureter, particularly where it crosses the uterine artery and lies in close proximity to the cervix. Ureteral injury may result from various mechanisms, including partial or complete transection by scalpel, inadvertent ligation with suture, or thermal injury—the latter being the most frequent cause. In minimally invasive procedures, the use of high-energy instruments for dissection, coagulation, and hemostasis further increases the risk of thermal damage to adjacent structures, particularly the ureter [1].

The incidence of ureteral injury during benign gynecological surgery is relatively low, estimated at approximately 0.08% [6]. The primary risk factor is endometriosis, which promotes fibrosis, distorts normal pelvic anatomy, and complicates surgical identification of the ureter—especially in proximity to the uterosacral ligaments [22]. In cases of deep infiltrating endometriosis, procedures such as ureterolysis and excision of endometriotic nodules near the ureter may be required, further elevating the risk of injury [7,55]. Regarding ureteral trauma during hysterectomy for benign conditions, the incidence varies depending on the surgical approach. Vaginal hysterectomy poses a risk due to limited visualization of the ureters, while laparoscopic hysterectomy is associated with the highest rate of ureteral injury (0.13%). This is followed by rates of 0.06% for both total vaginal and laparoscopic-assisted vaginal hysterectomy and 0.04% for total abdominal hysterectomy [22]. These findings were recently corroborated by a 2023 Cochrane review, which confirmed a greater risk of ureteral injury in laparoscopic hysterectomy for benign disease compared to the abdominal approach [56].

Similar to bladder injury, the incidence of iatrogenic ureteral damage is higher in hysterectomies performed for malignant conditions compared to those for benign pathologies, with reported rates ranging from 0.39% to 1.1% [11,26]. RH is technically demanding, and the risk of ureteral injury increases with the extent of lateral parametrial resection—particularly in cases of cervical carcinoma where wide excision of the parametrium is required [57]. Furthermore, surgical treatment of endometrial and cervical cancer often involves pelvic and/or para-aortic lymphadenectomy, during which ureteral identification and dissection are critical to avoid injury. In this context, the ureter serves as a key anatomical landmark, but its proximity to operative fields inherently increases the risk of iatrogenic trauma [58]. Minimally invasive radical hysterectomy—including both laparoscopic and robotic approaches—has been associated with a higher risk of ureteral injury compared to the open abdominal route [28]. However, a recent meta-analysis reported no significant differences in the incidence of ureteral or bladder complications between robotic and laparoscopic radical hysterectomy [59]. According to Kavallaris et al., pelvic and para-aortic lymph node dissection, although technically challenging, can be safely performed by surgical trainees under the supervision of an experienced surgeon, provided a standardized surgical approach is followed [60].

### 4.1. Diagnosis, Management, and Treatment

Ureteral injury is typically identified intraoperatively in approximately 8.6% of cases, with most diagnoses occurring postoperatively [61]. A key factor in this delay is the difficulty of detecting thermal injury during surgery. In fact, thermally induced urinary tract injuries are typically diagnosed after a mean interval of 12 days [62]. Clinical presentation may include hematuria, fever, signs of sepsis, elevated serum creatinine levels, and evidence of urinary tract obstruction [63]. The most accurate imaging modality for diagnosing ureteral injury is contrast-enhanced CT with a delayed excretory phase [29]. The hallmark radiologic finding is contrast extravasation from the ureter; additional signs may include urinoma formation and hydronephrosis. Retrograde or antegrade pyelography can further assist in diagnosis, offering the advantage of immediate therapeutic intervention, such as stent placement or percutaneous nephrostomy [31,32].

Management strategies depend on the timing of diagnosis and patient stability. Intraoperative recognition in a stable patient warrants immediate surgical repair. For unstable patients, temporary urinary diversion—typically via percutaneous nephrostomy—followed by delayed definitive repair is advised [31,32].

Furthermore, the choice of surgical approach is influenced by the location and extent of the injury, with different techniques indicated for proximal versus distal ureteral lesions and for short versus long segment defects [Table 3].

In cases of proximal and mid-ureteral injury involving short segments, primary uretero-ureterostomy can be performed laparoscopically [64]. When fibrosis or ischemia limits access to the renal pelvis, lower pole uretero-calycostomy is recommended [65]. For ureteral injuries exceeding 3 cm in length, transureteroureterostomy is often the preferred approach. In contrast, distal ureteral injuries are more prone to ischemic complications; thus, ureteroneocystostomy—which entails reimplantation of the ureter into the bladder—is the treatment of choice [66]. When the loss of ureteral length prevents a tension-free reimplantation, ureteroneocystostomy may be facilitated by a psoas hitch (anchoring the bladder to the ipsilateral psoas muscle) or a Boari flap (a bladder flap advancement technique) [67,68]. Thus, the gap caused by extensive loss of distal ureteral length can be bridged with a psoas hitch between the bladder and the ipsilateral psoas muscle or with a bladder flap (Boari flap). In extensive ureteral damage, ureteral substitution with an ileal interposition may be necessary [69]. Other reconstructive options include renal autotransplantation, where the kidney is relocated to the pelvis to shorten the ureteral course, and ureteroplasty using buccal mucosa grafts [70,71]. Traditionally, these reconstructive procedures have been performed using open surgical techniques. However, in recent decades, minimally invasive approaches—including both laparoscopic and robot-assisted methods—have demonstrated comparable success in managing complex ureteral injuries [72,73]. According to the European Association of Urology (EAU) guidelines, several key principles are essential for optimal ureteral reimplantation: meticulous debridement, ureteral spatulation, watertight mucosa-to-mucosa anastomosis, use of absorbable sutures, internal stenting, and isolation of the anastomotic site with peritoneum or an omental patch [32].

### 4.2. Ureterovaginal Fistula

A ureterovaginal fistula (UVF) is an abnormal communication between the ureter and the vaginal canal. Similar to a vesicovaginal fistula (VVF), the hallmark clinical symptom is continuous urinary leakage from the vagina, often detected through physical examination. As with VVF, contrast-enhanced CT urography remains the gold standard for diagnosis, as it enables visualization of contrast extravasation from the ureter into the vaginal vault [74]. Historically, the standard treatment for UVF was ureteroneocystostomy, which involves reimplantation of the ureter into the bladder [75]. However, management strategies have evolved in recent decades. Today, the first-line treatment for UVF is often ureteral stent placement, a minimally invasive and conservative approach with a high reported success rate [73,75]. When stenting fails or is contraindicated, surgical repair through ureteroneocystostomy remains a definitive option. This can be performed using a laparoscopic or robot-assisted approach and may be complemented by a psoas hitch or Boari flap to ensure a tension-free anastomosis in cases with extensive ureteral loss [74].

### 4.3. Prevention

The cornerstone of preventing iatrogenic ureteral injury during pelvic surgery is adequate visualization of the ureters. This requires the surgeon to possess a thorough understanding of pelvic anatomy, particularly the ureteral course. However, in certain cases, the ureter may deviate from its typical anatomical path due to factors such as previous surgeries, malignancies, endometriosis, or congenital variations. In such scenarios, surgical expertise becomes critical [76]. Gynecologic oncologic surgeons have extensive knowledge of the retroperitoneal space and are adept at navigating complex pelvic anatomy. The systematic opening of the paravesical and pararectal spaces facilitates ureteral identification and has been shown to significantly reduce the rate of urological complications [12,77,78]. Additionally, special attention must be given to the use of high-energy surgical devices, which may cause thermal injury to adjacent structures, including the ureter.

Prophylactic ureteral stenting may be beneficial, particularly during laparoscopic surgery, as it facilitates the tactile identification of the ureter and may thus help in detecting intraoperative ureteral damage. However, its role in preventing ureteral injury remains controversial. A recent review reported that prophylactic stenting significantly reduced the risk of ureteral injury in gynecologic surgeries, with a risk ratio of 0.44 (95% CI: 0.20–0.97; *p* = 0.04) [79]. Nevertheless, possible complications must be considered, including hematuria, urinary tract infection, and acute kidney injury [80,81].

An alternative technique for intraoperative ureteral identification involves the retrograde instillation of indocyanine green (ICG) via a ureteric catheter. This approach has demonstrated effectiveness in laparoscopic abdominal surgeries, particularly in gynecologic procedures [82,83,84]. More recently, a novel technique utilizing fluorescent ureteral catheters has been introduced as a means of enhancing intraoperative visualization [85,86]. Promising as it may be, this method is currently limited to clinical research settings.

Ultimately, effective prevention of iatrogenic ureteral injury depends on meticulous surgical technique and thorough knowledge of pelvic anatomy. While adjuncts like prophylactic stenting and fluorescence imaging may aid intraoperative visualization, their role is supportive. The surgeon’s expertise remains the most critical factor in preventing injury.

## 5. Innovations

In recent years, artificial intelligence (AI) has transitioned from diagnostic research to practical applications in gynecologic surgery, moving beyond theoretical models into intraoperative guidance and surgical education platforms. Numerous studies have explored its role in enhancing anatomical visualization and providing decision support. Both AI and augmented reality (AR) assist surgeons in the preoperative planning and intraoperative procedures by identifying and preserving critical anatomical structures [87].

A particularly promising area is the integration of AI with three-dimensional printing (3DP). By generating accurate 3D models of the surgical site, 3DP enables more detailed preoperative planning and improved spatial orientation during procedures, compared to conventional two-dimensional imaging [88]. This enhanced anatomical representation helps surgeons navigate the operative field more safely, thereby minimizing the risk to adjacent structures [88].

AR enhances surgical safety by overlaying critical anatomical information onto live laparoscopic images [89]. For instance, Bourdel et al. demonstrated AR’s use in laparoscopic myomectomy, providing a semi-transparent fusion of MRI-generated uterine and myoma models with endoscopic video, thereby improving tumor localization [90,91]. In an experimental study on pigs, AR increased ureter visibility from 31% to 81% and reduced localization error (~1.8 mm) [92,93], showcasing its potential in high-risk pelvic dissections. These systems remain preclinical or early clinical but demonstrate clear utility in complex anatomy identification

The combination of AI, AR, and 3DP has been shown to significantly reduce the incidence of iatrogenic injuries, including ureteral damage, in gynecologic surgery [91]. One notable example is a study by Feng Yu, who developed an AI-powered endoscope capable of estimating the ureter’s position and depth intraoperatively, achieving a mean identification accuracy of 92.04% [94]. More recently, research has focused on deep learning models capable of real-time ureteral auto-segmentation without the use of fluorescent agents, thus offering a non-invasive and efficient strategy for injury prevention [95].

Beyond intraoperative assistance, AI plays an increasingly important role in surgical skills training. Machine learning (ML), a subset of AI, is particularly valuable in this context, as it enables predictive analysis based on data patterns [96]. By incorporating variables such as motion trajectories, energy expenditure, and force application, ML algorithms can provide automated, quantitative assessments of surgical performance [97]. Such platforms generate real-time feedback, facilitate objective skill assessment and support personalized learning curves to enhance surgical safety [98].

Currently, these intraoperative AI-guided tools remain experimental or at the preclinical development stage, with most models evaluated only in simulation or early clinical settings and not yet approved for surgical use [99]. In contrast, AI-driven training tools are at a more advanced stage, with several validated machine-learning-based platforms already performing in clinical simulation environments. They offer structured, real-time assessment that supports individualized learning and promotes surgical proficiency prior to independent practice [100].

## 6. Discussion

This narrative review summarizes current evidence regarding the risk, diagnosis, prevention, and management of iatrogenic urinary tract injuries in gynecologic surgery [Table 4]. While the literature provides substantial insight into injury patterns and treatment strategies, the strength and consistency of evidence concerning preventive measures remain limited. Specifically, studies assessing prophylactic ureteral stenting [95,96], indocyanine green (ICG) instillation [84], and fluorescence-guided ureter visualization [85,86] and intraoperative cystoscopy [17,25,51,52,53,54] report heterogeneous and occasionally contradictory outcomes. This variability reflects ongoing debate regarding the routine use of these techniques in gynecologic practice, hindering the development of standardized protocols and highlighting the urgent need for more rigorous comparative studies. Major international societies, including the British Association of Urological Surgeons (BAUS), the American Urological Association (AUA), the European Association of Urology (EAU), and the World Society of Emergency Surgery (WSES), emphasize early identification and prevention of ureteral and bladder injuries. However, they concurrently acknowledge the limited availability of high-quality data supporting prophylactic interventions, reinforcing the need for further well-designed clinical research [5,29,31,32].

A notable limitation of this review is its narrative (non-systematic) design, which inherently carries a risk of selection bias. Nevertheless, it incorporates recent, high studies and reflects current clinical practice, including emerging strategies involving artificial intelligence (AI), augmented reality (AR), and three-dimensional printing (3DP) [87,88,89,90,91]. These technological innovations hold considerable promise for enhancing intraoperative decision-making, anatomical navigation, and surgical education. Preliminary data suggest that AI-assisted tools may reduce the incidence of iatrogenic urinary tract injuries by improving real-time visualization and anatomical recognition [93,95,96]. However, most of these technologies remain in the experimental or preclinical phase and require validation through prospective, multicenter trials. Furthermore, challenges related to cost, technical training, and equitable access must be addressed to facilitate broad and effective implementation [100].

## 7. Conclusions

Urinary tract injury is a rare but serious complication of pelvic surgery, most associated with gynecological procedures. Although infrequently fatal, such injuries can lead to significant physical and psychological morbidity. Therefore, prompt intraoperative recognition is essential for immediate repair and the reduction of long-term complications.

Several strategies have been developed to minimize the risk of urinary tract injury. Due to elevated risk in specific clinical scenarios, thorough preoperative risk assessment is recommended. Preventive measures include intraoperative cystoscopy, prophylactic ureteral stenting, indocyanine green instillation, and retroperitoneal dissection through the paravesical and pararectal spaces. Beyond technical interventions, the surgeon’s anatomical expertise and surgical experience remain paramount. Gynecologic surgeons trained in retroperitoneal anatomy are the best equipped to prevent urological complications in both benign and oncologic settings. Notably, lower rates of urinary tract injury have been observed in procedures performed by high-volume or specialized surgeons.

Artificial intelligence (AI) is emerging as a promising tool to reduce urinary tract injuries by enhancing real-time ureter identification through integration with augmented reality and 3D printing. AI also supports surgical training by providing automated skill assessment, promoting safer and more effective surgeries. Although most AI applications are still experimental, they hold significant potential to improve surgical outcomes and reduce complications. To appreciate the clinical benefits of these technologies, AI-assisted imaging and navigation systems should be systematically integrated into gynecologic surgical practice, particularly in high-risk or complex procedures. Concurrently, standardized training programs must be developed to ensure safe and effective use of these technologies. From a policy perspective, healthcare systems should prioritize investment in surgical innovation, particularly in digital infrastructure, AI tools, and surgeon education. Guidelines should be updated to reflect emerging evidence and incorporate risk-stratified recommendations for the prevention and early detection of urinary tract injuries. Moreover, centralization of complex gynecologic surgeries to specialized centers may further decrease complication rates. Future research should focus on the validation and cost-effectiveness of new preventive strategies, including AI-driven approaches, and explore ways to ensure equitable access to these innovations across different healthcare settings.

## Figures and Tables

**Table 1:** Incidence of urinary injury during surgery. The table summarizes the reported incidence rates of urinary tract injuries across different surgical specialties. Gynecological procedures account for the highest proportion of injuries, followed by urological and colorectal surgeries.

Type of Surgery	Rates
Gynecological procedures	50%
Urological procedures	30%
Colorectal procedures	5–15%

**Table 2:** Goh’s classification of genitourinary fistula. This system classifies genitourinary fistulas by fistula length (distance from the external urinary meatus), size (largest diameter), and degree of vaginal scarring. These factors guide surgical planning and predict repair outcomes.

Feature	Classification	Description
Length	Type 1	Distal edge of fistula > 3.5 cm from external urinary meatus
	Type 2	Distal edge of fistula 2.5–3.5 cm from external urinary meatus
	Type 3	Distal edge of fistula 1.5–<2.5 cm from external urinary meatus
	Type 4	Distal edge of fistula < 1.5 cm from external urinary meatus
Size	a	<1.5 cm, in the largest diameter
	b	1.5–3 cm, in the largest diameter
	c	>3 cm, in the largest diameter
Vaginal scarring	i.	No or mild fibrosis around the fistula/vagina and/or vaginal length > 6 cm or normal capacity
	ii.	Moderate or severe fibrosis around the fistula and/or vagina and/or reduced vaginal length
	iii.	Special considerations, e.g., circumferential fistula, involvement of ureteric orifices

**Table 3:** Management of ureteral injury based on anatomical location. Treatment varies by injury site: proximal and middle third lesions are typically managed with end-to-end anastomosis or transureteroureterostomy; distal injuries often require a psoas hitch; long-segment defects may need grafts, intestinal interposition, or renal autotransplantation.

Type of Injury	Treatment
Proximal one-third	- End-to-end anastomosis - Tranureteroureterostomy - Ureterocalycostomy
Middle one-third	- End-to-end anastomosis - Tranureteroureterostomy - Boari flap
Distal one-third	- Psoas Hitch
Long segment	- Oral graft ureteroplasty - Intestinal interposition - Autotransplant

**Table 4:** Comparative summary. This table provides a side-by-side overview of the two most common urological injuries—bladder and ureteral—that may occur during gynecologic surgical procedures.

Injury Type	Incidence	High-Risk Procedures	Main Consequences	Diagnosis
Bladder	0.24–3.7%	Radical hysterectomy, vaginal hysterectomy	Fistulas, hematuria, infection, delayed recovery	85% identified intraoperatively
Ureter	0.08–1.1%	Radical hysterectomy, laparoscopic hysterectomy	Fistulas, hydronephrosis, sepsis, urinoma	8.6% identified intraoperatively

## Data Availability

Not applicable.

## Abbreviations

The following abbreviations are used in this manuscript:

RH	Radical hysterectomy
VVF	Vesicovaginal fistula
UVF	Ureterovaginal fistula
ICG	Indocyanine green
AI	Artificial intelligence
3DP	Three-dimensional printing
AR	Augmented reality
